# Supplementary material for: OSMR controls glioma stem cell respiration and confers resistance of glioblastoma to ionizing radiation
Source: Nat Commun. 2020 Aug 17;11:4116. doi: 10.1038/s41467-020-17885-z (PMC7431428; doi:10.1038/s41467-020-17885-z)
Supplement: Supplementary file 1 — Supplementary Information [file 41467_2020_17885_MOESM1_ESM.pdf]

Supplementary Information

OSMR controls glioma stem cell respiration and confers resistance of glioblastoma to ionizing radiation

Sharanek, Burban et al.

Supplementary Table. 1. IP-LC-MS/MS screen reveals potential OSMR binding partners in the mitochondria.

EGFRvIII-expressing astrocytes were subjected to endogenous IP using antibodies to OSMR or an IgG control. Pull downs were analyzed using LC-MS/MS. This table represents the potential OSMR binding partners in the mitochondria.

Accession	Protein Name
MCCA_MOUSE	Methylcrotonoyl-CoA carboxylase subunit alpha, mitochondrial OS=Mus musculus GN=Mccc1 PE=2 SV=2
PYC_MOUSE	Pyruvate carboxylase, mitochondrial OS=Mus musculus GN=Pc PE=1 SV=1
PCCA_MOUSE	Propionyl-CoA carboxylase alpha chain, mitochondrial OS=Mus musculus GN=Pcca PE=2 SV=2
Q5SWU9|COA1_MOUSE	Acetyl-CoA carboxylase 1 - Mus musculus (Mouse)
GRP75_MOUSE	Stress-70 protein, mitochondrial OS=Mus musculus GN=Hspa9 PE=1 SV=2
CISY_MOUSE	Citrate synthase, mitochondrial OS=Mus musculus GN=Cs PE=1 SV=1
LONM_MOUSE	Lon protease homolog, mitochondrial OS=Mus musculus GN=Lonp1 PE=1 SV=2
ETFA_MOUSE	Electron transfer flavoprotein subunit alpha, mitochondrial OS=Mus musculus GN=Etfa PE=1 SV=2
M2OM_MOUSE	Mitochondrial 2-oxoglutarate/malate carrier protein OS=Mus musculus GN=Slc25a11 PE=1 SV=3
ODPA_MOUSE	Pyruvate dehydrogenase E1 component subunit alpha, somatic form, mitochondrial OS=Mus musculus GN=Pdha1 PE=1 SV=1
ADCK3_MOUSE	Chaperone activity of bc1 complex-like, mitochondrial OS=Mus musculus GN=Adck3 PE=2 SV=2
EFTS_MOUSE	Elongation factor Ts, mitochondrial OS=Mus musculus GN=Tsfm PE=2 SV=1
ECHM_MOUSE	Enoyl-CoA hydratase, mitochondrial OS=Mus musculus GN=Echs1 PE=1 SV=1
A2APD8|A2APD8_MOUSE	Isocitrate dehydrogenase 3 - Mus musculus (Mouse)
Q3TIT9_MOUSE	Acetyl-Coenzyme A acyltransferase 2 (Mitochondrial 3-oxoacyl-Coenzyme A thiolase), isoform CRA_k OS=Mus musculus GN=Acaa2 PE=2 SV=1
Q2NLC5_MOUSE	Timm44 protein OS=Mus musculus GN=Timm44 PE=2 SV=1
ACADL_MOUSE	Long-chain specific acyl-CoA dehydrogenase, mitochondrial OS=Mus musculus GN=Acadl PE=2 SV=2
ES1_MOUSE	ES1 protein homolog, mitochondrial OS=Mus musculus GN=D10Jhu81e PE=1 SV=1
FUMH_MOUSE	Fumarate hydratase, mitochondrial OS=Mus musculus GN=Fh PE=1 SV=2

CMC2_MOUSE	Calcium-binding mitochondrial carrier protein Aralar2 OS=Mus musculus GN=Slc25a13 PE=1 SV=1
Q3TBZ2|Q3TBZ2_MOUSE	NOD-derived CD11c +ve dendritic cells cDNA, RIKEN full-length enriched library, clone:F630215F23 product:translocase of outer mitochondrial membrane 40 homolog - Mus musculus (Mouse)
SUCA_MOUSE	Succinyl-CoA ligase [GDP-forming] subunit alpha, mitochondrial OS=Mus musculus GN=Suclg1 PE=1 SV=4
VDAC2_MOUSE	Voltage-dependent anion-selective channel protein 2 OS=Mus musculus GN=Vdac2 PE=1 SV=2
ALDH2_MOUSE	Aldehyde dehydrogenase, mitochondrial OS=Mus musculus GN=Aldh2 PE=1 SV=1
A2AIW8_MOUSE	Peptidase (Mitochondrial processing) alpha OS=Mus musculus GN=Pmpca PE=3 SV=1
ECHB_MOUSE	Trifunctional enzyme subunit beta, mitochondrial OS=Mus musculus GN=Hadhb PE=1 SV=1
DHE3_MOUSE	Glutamate dehydrogenase 1, mitochondrial OS=Mus musculus GN=Glud1 PE=1 SV=1
THIL_MOUSE	Acetyl-CoA acetyltransferase, mitochondrial OS=Mus musculus GN=Acat1 PE=1 SV=1
A2AWH6_MOUSE	Pyruvate dehydrogenase complex, component X OS=Mus musculus GN=Pdhx PE=3 SV=1
ACDSB_MOUSE	Short/branched chain specific acyl-CoA dehydrogenase, mitochondrial OS=Mus musculus GN=Acadsb PE=1 SV=1
BCS1_MOUSE	Mitochondrial chaperone BCS1 OS=Mus musculus GN=Bcs1l PE=1 SV=1
DHSA_MOUSE	Succinate dehydrogenase [ubiquinone] flavoprotein subunit, mitochondrial OS=Mus musculus GN=Sdha PE=1 SV=1
NDUS1_MOUSE	NADH-ubiquinone oxidoreductase 75 kDa subunit, mitochondrial OS=Mus musculus GN=Ndufs1 PE=1 SV=1
NDUS2_MOUSE	NADH dehydrogenase [ubiquinone] iron-sulfur protein 2, mitochondrial OS=Mus musculus GN=Ndufs2 PE=1 SV=1
NNTM_MOUSE	NAD(P) transhydrogenase, mitochondrial OS=Mus musculus GN=Nnt PE=1 SV=2
P70404|IDH3G_MOUSE	Isocitrate dehydrogenase [NAD] subunit gamma, mitochondrial precursor - Mus musculus (Mouse)
Q9DB77|UQCR2_MOUSE	Ubiquinol-cytochrome-c reductase complex core protein 2, mitochondrial precursor - Mus musculus (Mouse)
SYAM_MOUSE	Alanyl-tRNA synthetase, mitochondrial OS=Mus musculus GN=Aars2 PE=2 SV=1

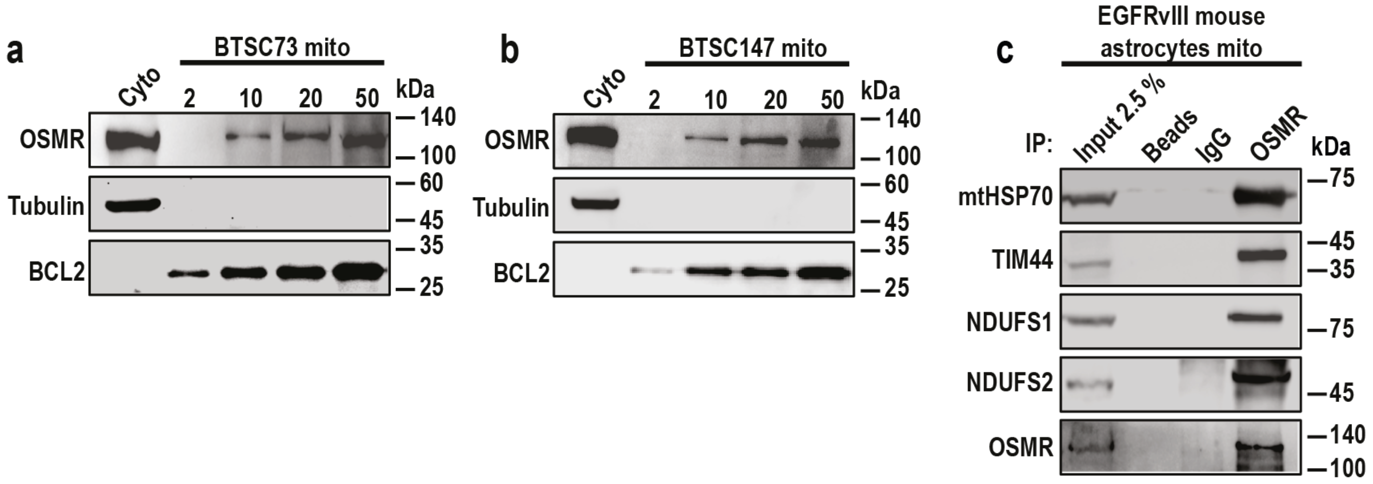

Supplementary Fig. 1. OSMR is targeted to the mitochondria.

(**a-b**) Different concentrations of mitochondrial fractions were analyzed in BTSC73 (**a**) and BTSC147 (**b**). α-Tubulin and BCL2 were used as controls for cytoplasmic and mitochondrial fractions, respectively. Cyto: Cytoplasmic; Mito: mitochondrial. (**c**) Mitochondrial fractions from EGFRvIII-expressing mouse astrocytes were subjected to immunoprecipitation using antibodies to OSMR or mouse IgG control followed by immunoblotting with antibodies to mtHSP70, TIM44, NDUFS1, NDUFS2 or OSMR. The blots for each of the cell lines are representative of three replicates performed on different passage numbers.

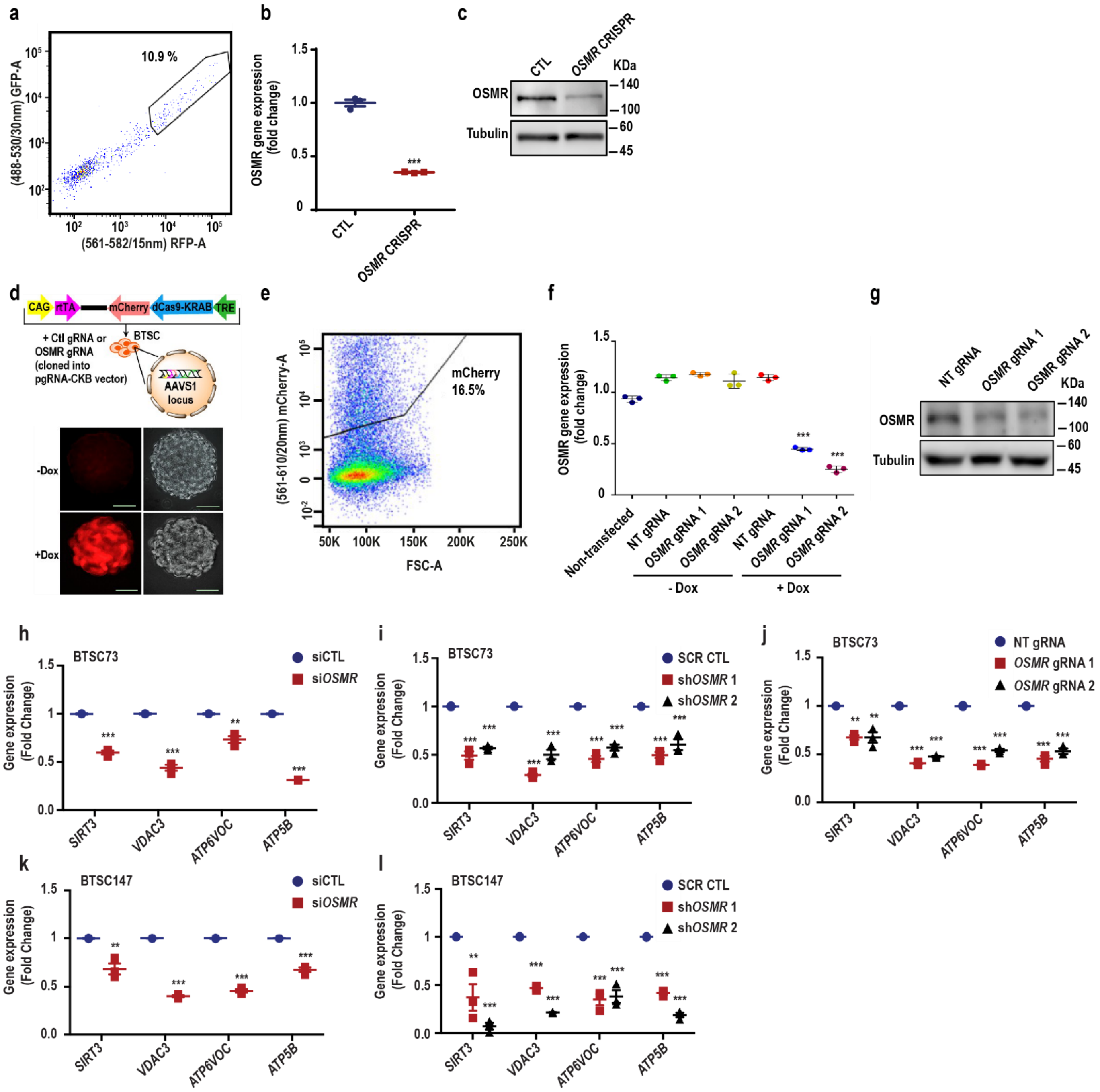

Supplementary Fig. 2. Validation of metabolic/mitochondrial candidate target genes in patient-derived *OSMR* KD BTSCs.

(**a**) Single-cell FACS sorting by gating on a live population of double GFP and RFP positive BTSC73 cells was performed to obtain cells that received both gRNA to *OSMR* gene. (**b-c**) *OSMR* mRNA (**b**) and protein (**c**) expression levels were evaluated by RT-qPCR (***p < 0.0001; Unpaired two-tailed t-test, n = 3), or immunoblotting analyses, respectively. The Western blot represents three replicates

performed on different BTSC passage number. (**d-g**) *OSMR* KD of BTSC73 was generated using dox-inducible CRISPR/dCas9 (CRISPRi). The expression of dCas9 was verified after 10 days of doxycycline (Dox) induction by visualizing mCherry-expressing cells (**d**). FACS sorting by gating on a live population of mCherry positive CRISPRi BTSC73 cells was performed to obtain a stable mCherry expressing population (**e**). *OSMR* gene (**f**) and protein (**g**) expression was evaluated in CRISPRi BTSC73 in the absence and presence of Dox by RT-qPCR (***$p < 0.0001$; Unpaired two-tailed t-test, n = 3) or immunoblotting. Western blot represents a minimum of three replicates from different BTSC passage number. (**h-l**) mRNA expression levels of *ATP5b*, *SIRT3*, *ATP6VOC*, and *VDAC3* were quantified by RT-qPCR in *OSMR* KD BTSCs. BTSC73 electroporated with either si*OSMR* or siCTL (**h**): ***$p < 0.0001$ for each pairwise comparison except: **$p_{ATP6VOC} = 0.0017$; Unpaired two-tailed t-test, n = 3; BTSC73 transduced with sh*OSMR* or SCR CTL lentiviruses (**i**): ***$p < 0.0001$ for each pairwise comparison except: ***p_{ATP5b} (SCR CTL vs. sh*OSMR* 1) = 0.0001, ***p_{ATP5b} (SCR CTL vs. sh*OSMR 2*) = 0.0005; One-way ANOVA followed by Dunnett's test for multiple comparisons, n = 3; BTSC73 transduced with non-targeting (NT) or *OSMR* gRNA lentiviruses (**j**): ***$p < 0.0001$ for each pairwise comparison except: **$p_{SIRT3} = 0.0010$; One-way ANOVA followed by Dunnett's test for multiple comparisons, n = 3; BTSC147 electroporated with either si*OSMR* or siCTL (**k**): ***$p < 0.0001$ for each pairwise comparison except: **$p_{SIRT3} = 0.0052$, ***$p_{ATP5b} = 0.0002$; Unpaired two-tailed t-test, n = 3; BTSC147 transduced with sh*OSMR* or SCR CTL lentiviruses (**l**): ***$p < 0.0001$ for each pairwise comparison except: **p_{SIRT3} (SCR CTL vs. sh*OSMR* 1) = 0.0029, ***$p_{ATP6VOC}$ (SCR CTL vs. sh*OSMR* 1) = 0.0002, ***p_{SIRT3} (SCR CTL vs. sh*OSMR* 2) = 0.0004, ***$p_{ATP6VOC}$ (SCR CTL vs. sh*OSMR* 2) = 0.0003. Data are presented as the mean ± SEM, n = 3 independent biological samples.

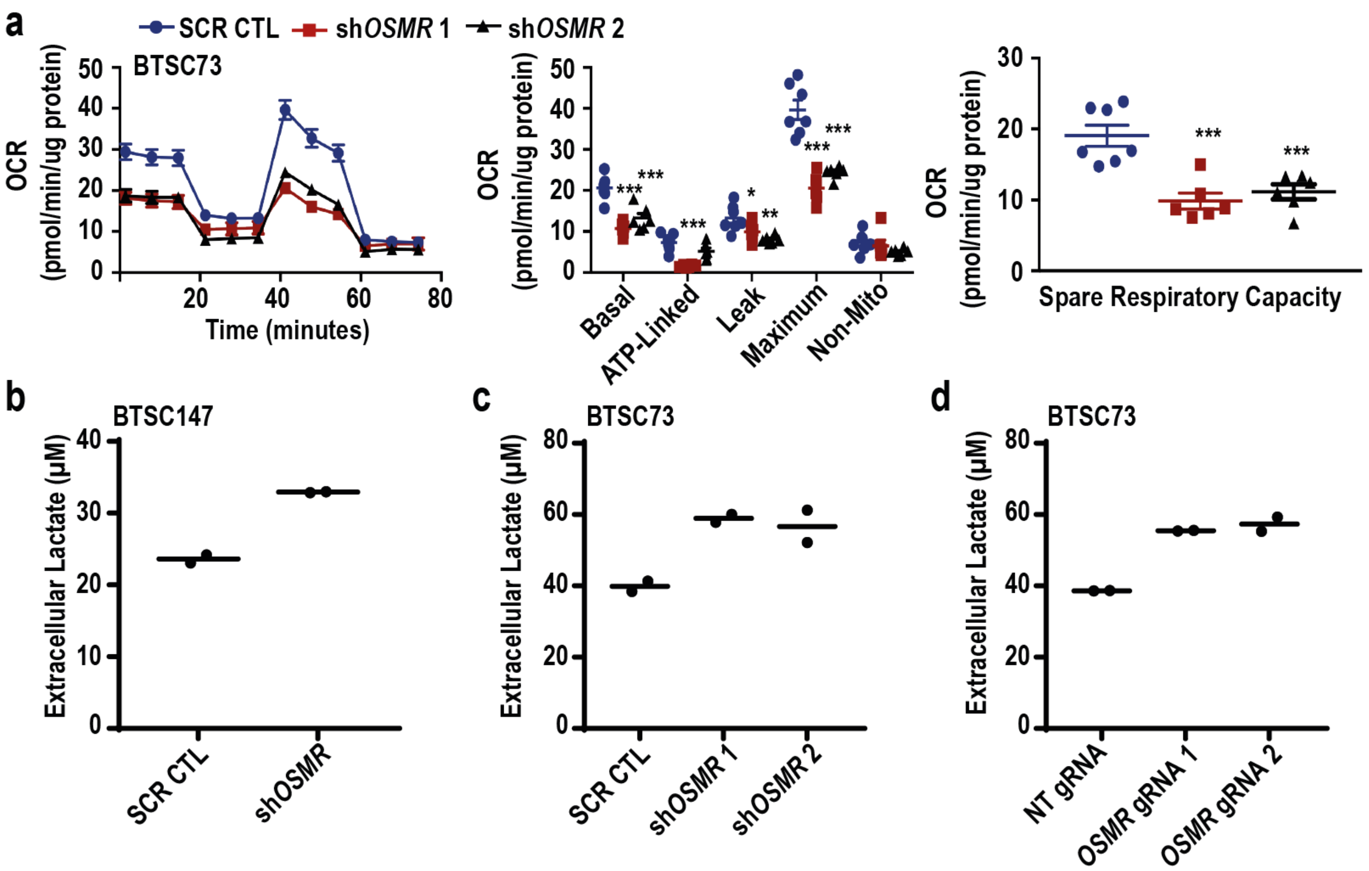

Supplementary Fig. 3. *OSMR* KD impairs OCR and increases extracellular lactate in BTSCs. (**a**) BTSC73 transduced with *OSMR* shRNA1/2 or scramble shRNA control (SCR CTL), were subjected to bioenergetic analysis using a Seahorse XFe96 Bioenergetic Flux Analyzer. OCR are plotted (left panel). Data is plotted to demonstrate the differences between basal, ATP-linked, proton leak, maximal, and non-mitochondrial respiration (middle panel). Spare respiratory capacity (SRC) is plotted (right panel). ***$p < 0.0001$ for each pairwise comparison except: *p_{Leak} (SCR vs. sh*OSMR* 1) = 0.0426, **p_{Leak} (SCR vs. sh*OSMR* 2) = 0.0023, ***p_{Basal} (SCR vs. sh*OSMR* 1) = 0.0001, ***p_{Basal} (SCR vs. sh*OSMR* 2) = 0.0002, ***p_{SRC} (SCR vs. sh*OSMR* 1) = 0.0002, ***p_{SRC} (SCR vs. sh*OSMR* 2) = 0.0008; One-way ANOVA followed by Dunnett's test, n ≥ 6 independent biological samples. Data are presented as the mean ± SEM. (**b-d**) Extracellular lactate levels were measured in *OSMR* KD BTSC147(**b**), *OSMR* KD BTSC73 (**c**) or *OSMR* CRISPRi BTSC73 (**d**). Data are presented as the mean, n = 2 independent biological samples.

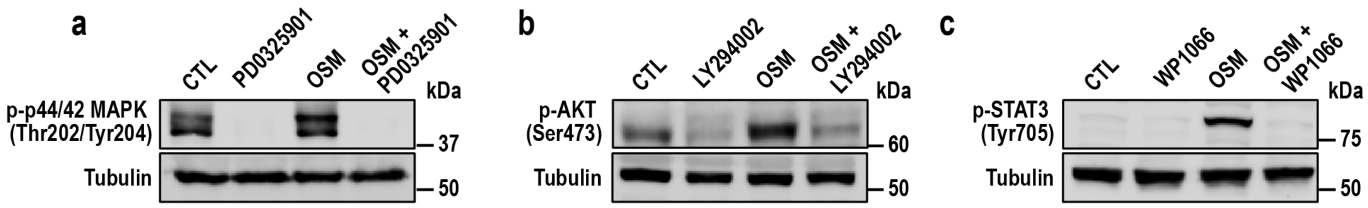

Supplementary Fig. 4. PI3K/AKT, MAPK/ERK and JAK/STAT signalling pathways are activated in response to OSM in BTSCs.

(**a-c**) BTSC73 were pre-incubated with 10 ng/mL of OSM for 24 h followed by incubation of cells for 2 h with either of 10 µM of MAPK/ERK inhibitor, PD0325901 (**a**), 10 µM of PI3K/AKT inhibitor, LY294002 (**b**) or 20 µM of JAK/STAT3 inhibitor, WP1066 (**c**). The cells were lysed and immunoblotted with the indicated antibodies. α-Tubulin was used as loading control. The blots represent a minimum of three replicates from different BTSC passage numbers.

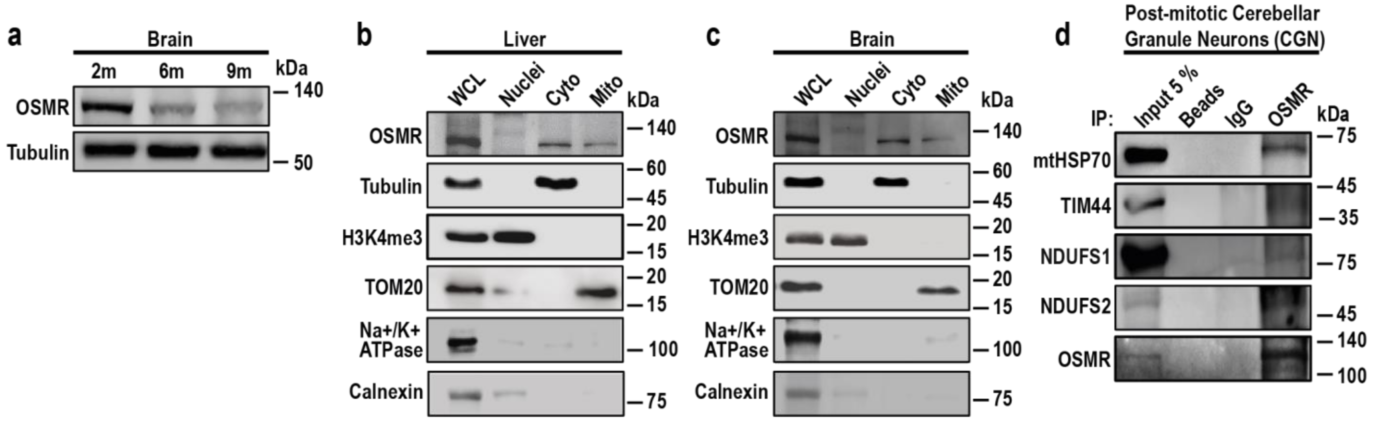

Supplementary Fig. 5. Presence of OSMR in the mitochondria.

(**a**) OSMR expression levels in the brains of 2-, 6-, and 9-month-old mice were analyzed by immunoblotting using an OSMR antibody. α-Tubulin was used as a loading control. The Western blot represents a minimum of three biological replicates. (**b-c**) Cell fractionations were performed in liver (**b**) and brain (**c**) tissues in adult mice (8 weeks old). Cell lysates were analyzed by immunoblotting using indicated antibodies as described in Fig. 1a-d. WCL: Whole-cell lysates; Cyto: cytoplasmic; Mito: mitochondrial. The Western blots represent a minimum of three biological replicates. (**d**) OSMR-overexpressing primary CGN cultures were subjected to immunoprecipitation using antibodies to OSMR or mouse IgG control, followed by immunoblotting with the indicated antibodies. The IP-Western blots represent a minimum of three replicates from independent CGN cultures.

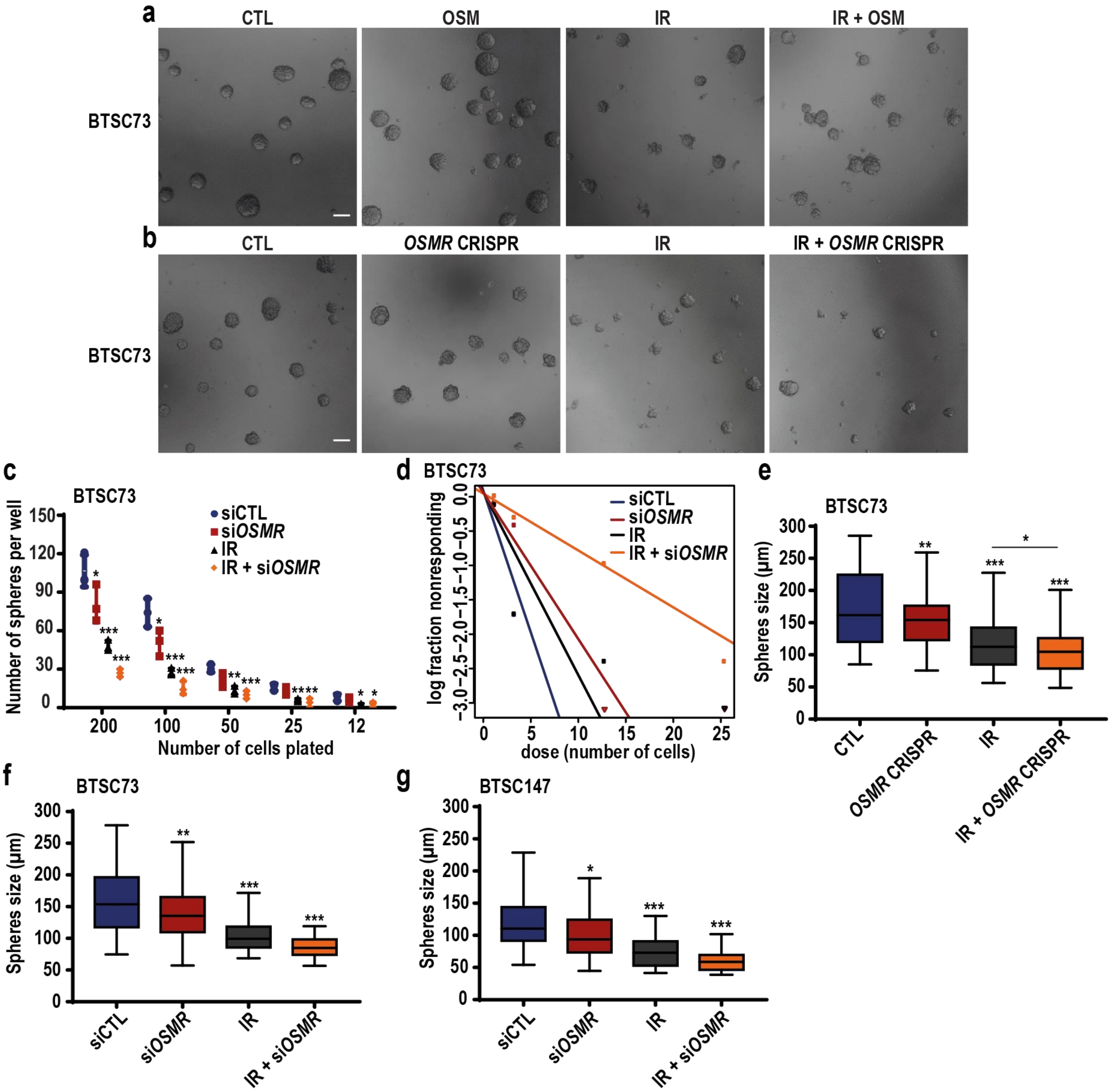

Supplementary Fig. 6. OSM/OSMR confers resistance of BTSCs to IR.

(**a-b**) Representative phase-contrast images of three different cultures of irradiated (4 Gy) BTSC73 in the presence and absence of OSM (10 ng/mL) (**a**) and in *OSMR* CRISPR and control BTSC73 (**b**) are shown. Images were taken following 7 days of plating. Scale bar = 200 μm. (**c**) LDA was performed on BTSC73 electroporated with si*OSMR* or siCTL in the absence and presence of 4 Gy of IR. All the counts were performed following 7 days of plating. ***$p < 0.0001$ for each pairwise comparison except: 200

cells (*$p_{siCTL\ vs.\ si\mathit{OSMR}}$ = 0.044, ***$p_{siCTL\ vs.\ IR}$ = 0.0004), 100 cells (*$p_{siCTL\ vs.\ si\mathit{OSMR}}$ = 0.0297, ***$p_{siCTL\ vs.\ IR}$ = 0.0005), 50 cells (**$p_{siCTL\ vs.\ IR}$ = 0.0027, ***$p_{siCTL\ vs.\ IR\ +\ si\mathit{OSMR}}$ = 0.0007), 25 cells (**$p_{siCTL\ vs.\ IR}$ = 0.0095, **$p_{siCTL\ vs.\ IR\ +\ si\mathit{OSMR}}$ = 0.0027), 12 cells (*$p_{siCTL\ vs.\ IR}$ = 0.0266, *$p_{siCTL\ vs.\ IR\ +\ si\mathit{OSMR}}$ = 0.0454); One-way ANOVA followed by Tukey's test for multiple comparisons, n = 3 independent biological cell cultures. Data are presented as the mean ± SEM. (**d**) ELDA was performed on BTSC73 electroporated with si*OSMR* or siCTL in the absence and presence of 4 Gy of IR. (**e-g**) Size of spheres was measured in *OSMR* KD BTSCs 7 days following 4 Gy of IR. *OSMR* CRISPR and control BTSC73 (**e**): ***p < 0.0001 for each pairwise comparison except: *$p_{IR\ vs.\ IR\ +\ \mathit{OSMR}\ CRISPR}$ = 0.0476, **$p_{CTL\ vs.\ \mathit{OSMR}\ CRISPR}$ = 0.0098; One-way ANOVA followed by Tukey's test for multiple comparisons; BTSC73 electroporated with si*OSMR* or siCTL (**f**): ***p < 0.0001 for each pairwise comparison except: **$p_{siCTL\ vs.\ si\mathit{OSMR}}$ = 0.004; One-way ANOVA followed by Tukey's test for multiple comparisons; BTSC147 electroporated with si*OSMR* or siCTL (**g**): ***p < 0.0001 for each pairwise comparison except: *$p_{siCTL\ vs.\ si\mathit{OSMR}}$ = 0.0205; One-way ANOVA followed by Tukey's test for multiple comparisons. Size of spheres of 3 independent biological cell cultures are presented as box plots showing 25th and 75th percentiles (box), median (center line), minima and maxima (whiskers).

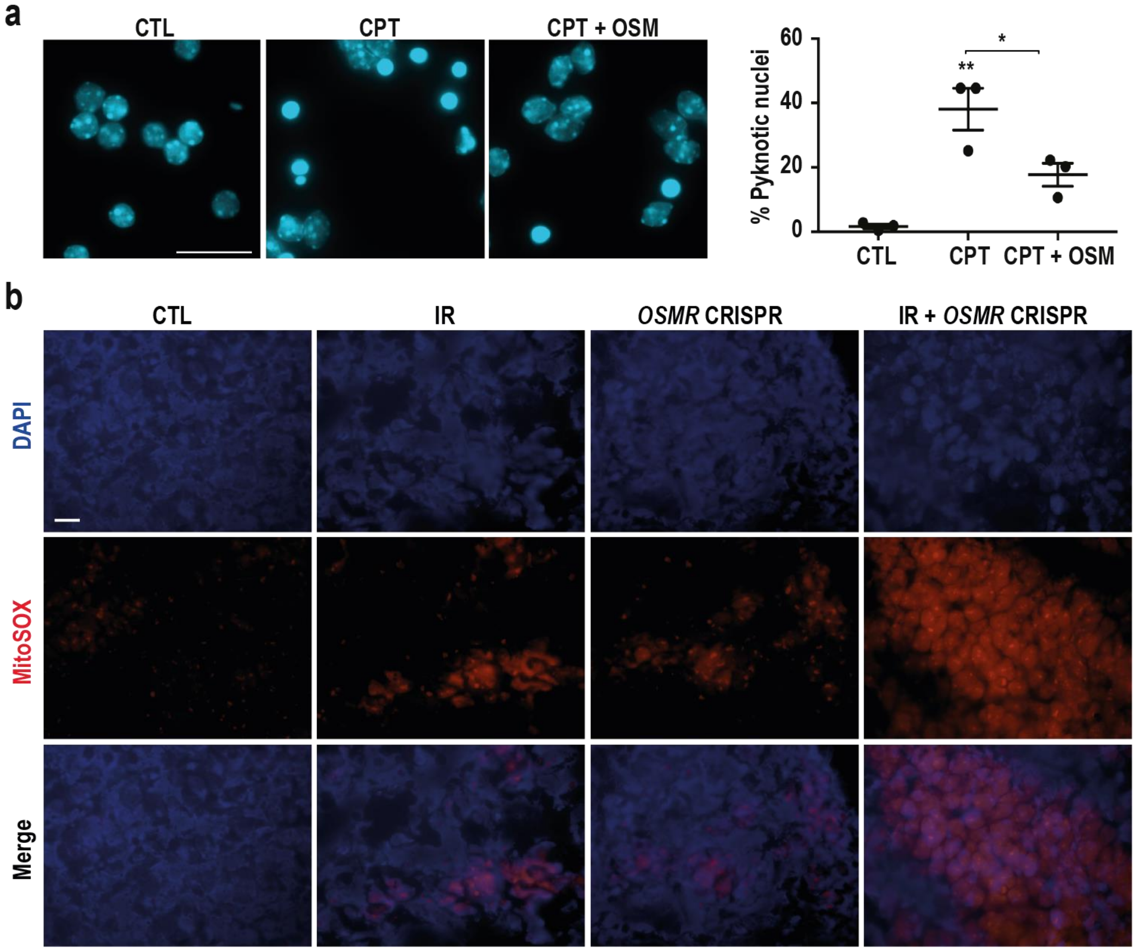

Supplementary Fig. 7. OSM/OSMR regulates ROS production and confers resistance to DNA damage-induced cell death.

(a) OSMR-overexpressing primary CGN were treated with camptothecin (CPT) following 3 h of incubation with 100 ng/mL of OSM. Neurons were stained with DAPI following 16 h of treatment with CPT. Representative images of 3 independent biological cell cultures are shown. Scale bar = 20 µm. For each replicate, approximately 500 nuclei were scored for each sample. $*p_{\text{CPT vs. CPT + OSM}} = 0.0351$, $**p_{\text{CTL vs. CPT}} = 0.0023$; One-way ANOVA followed by Dunnett's test for multiple comparisons, n = 3 independent biological cell cultures. Data are presented as the mean ± SEM. **(b)** *OSMR* CRISPR and control BTSC73 were subcutaneously injected into the right flank of Fox Chase SCID mice, followed by treatment with 4 Gy of IR. Non-irradiated tumours were used as control. Sections from IR treated and non-irradiated tumours were stained with MitoSOX. Representative images of 4 different tumour sections are shown. Scale bar = 20 µm.

Fig. 1a

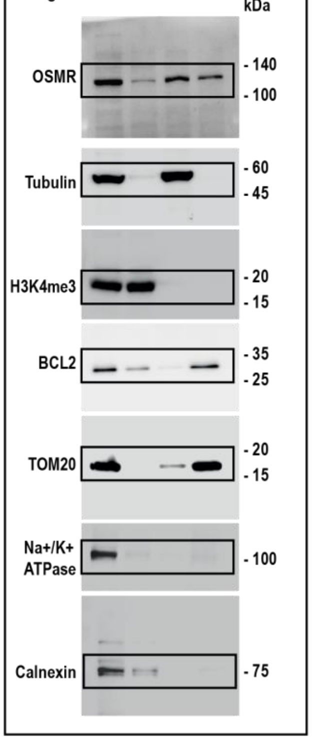

Fig. 1b

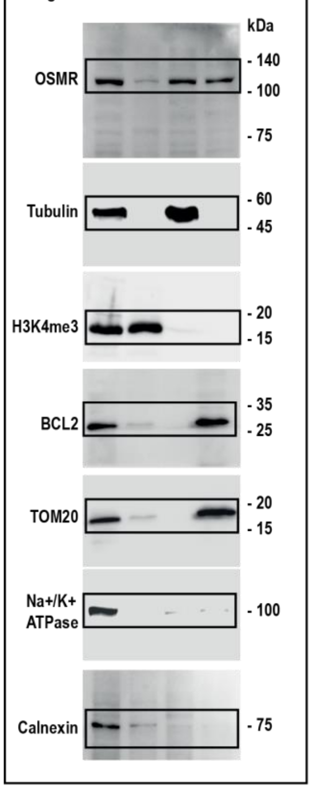

Fig. 1c

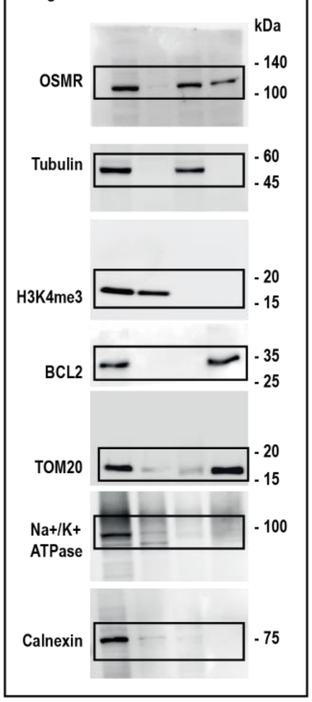

Fig. 1d

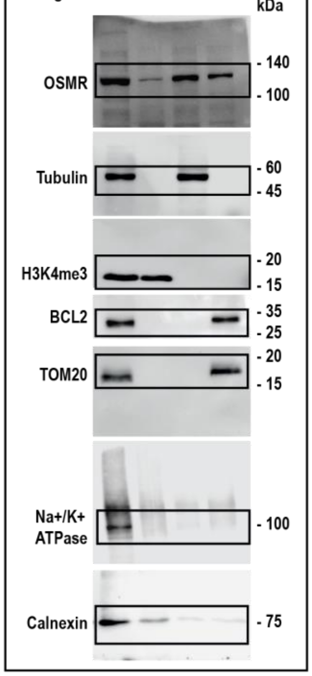

Fig. 2a

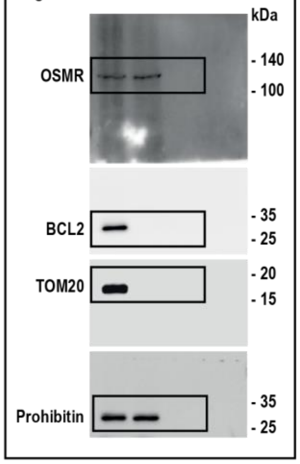

Fig. 2b

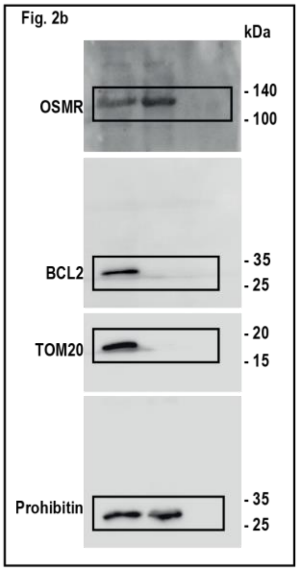

Fig. 2c

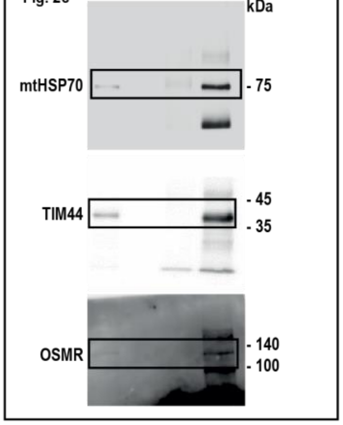

Fig. 2d

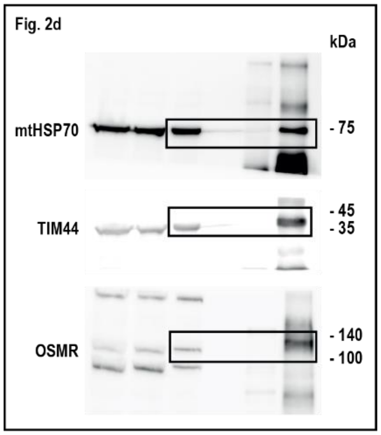

Fig. 2e

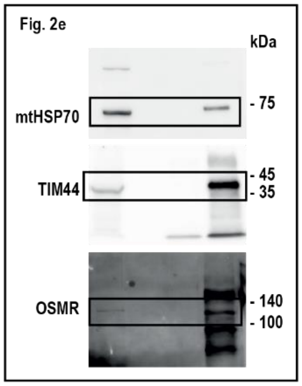

Fig. 2f

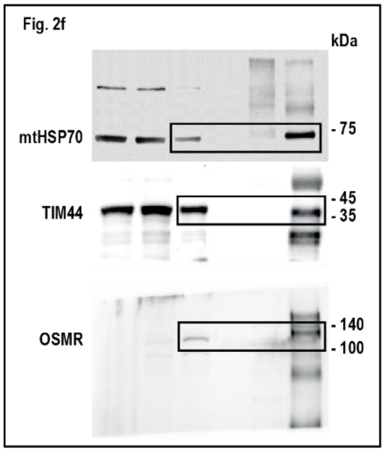

Fig. 2j

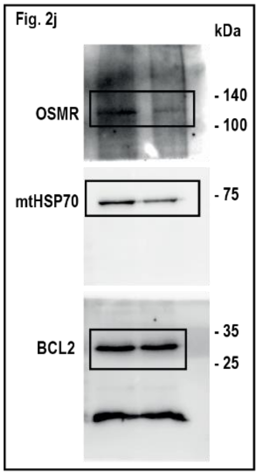

Fig. 2k

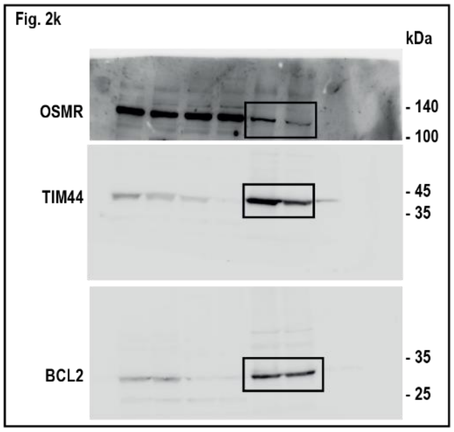

Fig. 2l

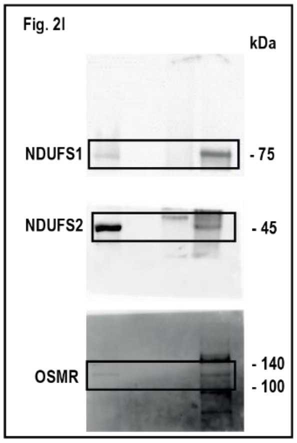

Fig. 2m

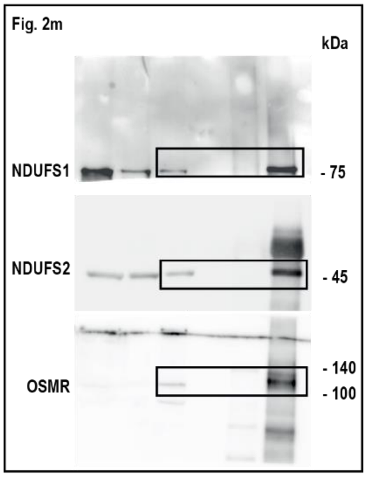

Fig. 2n

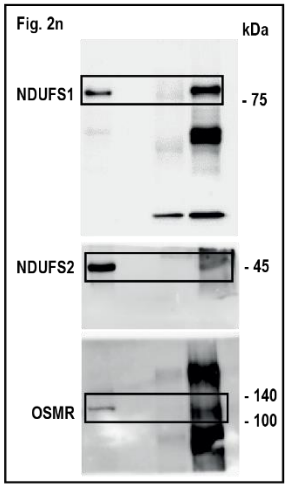

Fig. 2o

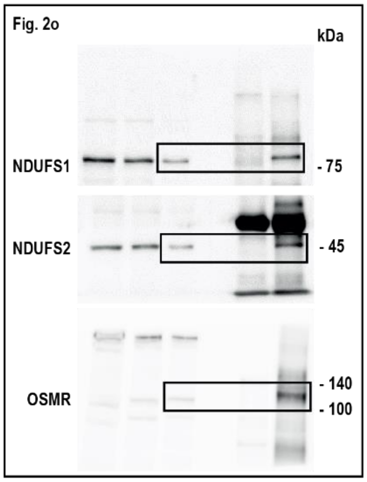

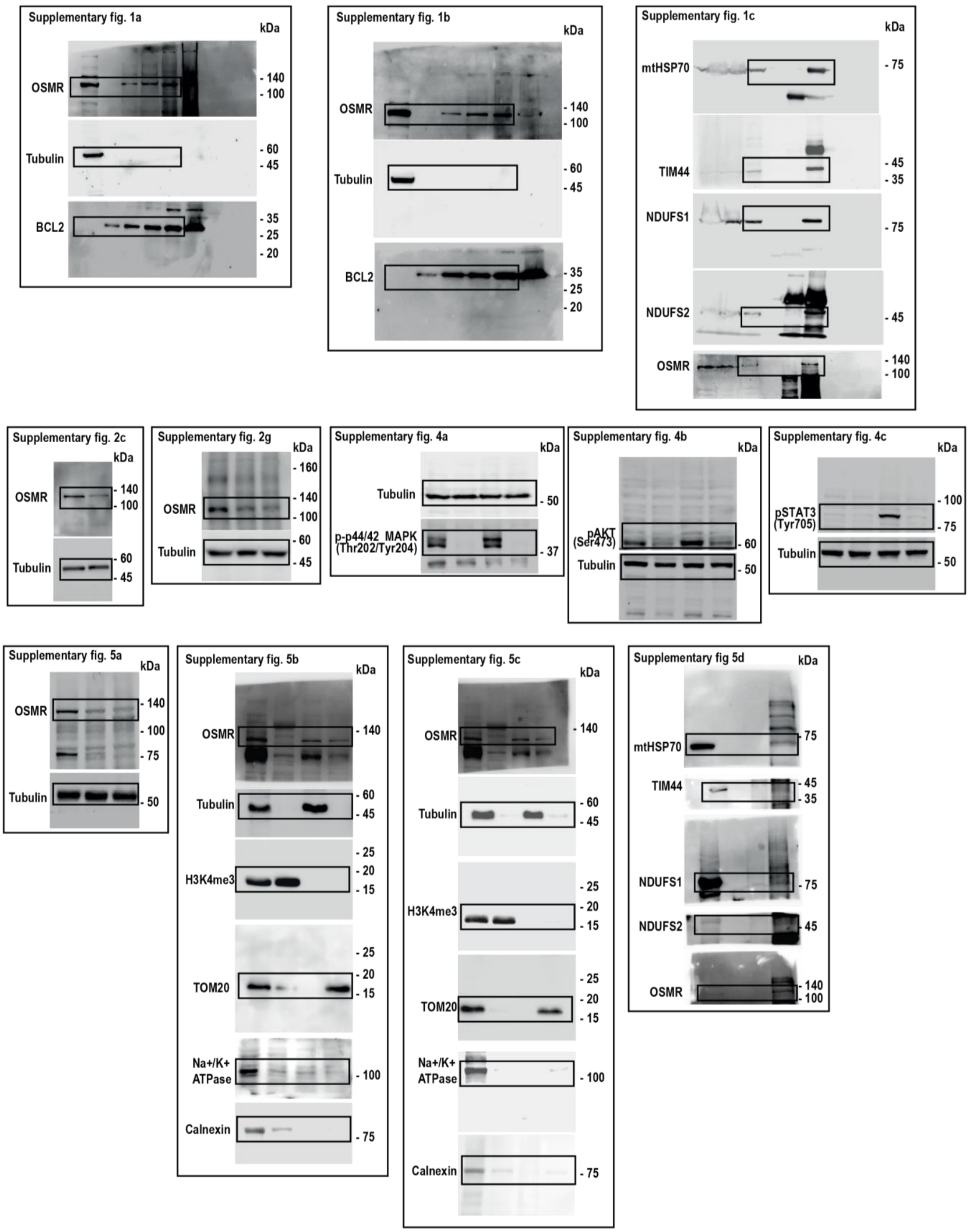

Supplementary Fig. 8. Original immunoblots.

Uncropped images of immunoblots are shown in the order they are presented in the figures.
