# Supplementary material for: OSMR controls glioma stem cell respiration and confers resistance of glioblastoma to ionizing radiation
Source: Nat Commun. 2020 Aug 17;11:4116. doi: 10.1038/s41467-020-17885-z (PMC7431428; doi:10.1038/s41467-020-17885-z)
Supplement: Supplementary file 3 — Reporting Summary [file 41467_2020_17885_MOESM3_ESM.pdf]

nature research

Corresponding author(s): Arezu Jahani-Asl

Last updated by author(s): Jun 26, 2020

Reporting Summary

Nature Research wishes to improve the reproducibility of the work that we publish. This form provides structure for consistency and transparency in reporting. For further information on Nature Research policies, see our Editorial Policies and the Editorial Policy Checklist.

Statistics

For all statistical analyses, confirm that the following items are present in the figure legend, table legend, main text, or Methods section.

n/a	Confirmed	
☐	☒	The exact sample size (*n*) for each experimental group/condition, given as a discrete number and unit of measurement
☐	☒	A statement on whether measurements were taken from distinct samples or whether the same sample was measured repeatedly
☐	☒	The statistical test(s) used AND whether they are one- or two-sided<br>*Only common tests should be described solely by name; describe more complex techniques in the Methods section.*
☐	☒	A description of all covariates tested
☐	☒	A description of any assumptions or corrections, such as tests of normality and adjustment for multiple comparisons
☐	☒	A full description of the statistical parameters including central tendency (e.g. means) or other basic estimates (e.g. regression coefficient) AND variation (e.g. standard deviation) or associated estimates of uncertainty (e.g. confidence intervals)
☐	☒	For null hypothesis testing, the test statistic (e.g. *F*, *t*, *r*) with confidence intervals, effect sizes, degrees of freedom and *P* value noted<br>*Give P values as exact values whenever suitable.*
☒	☐	For Bayesian analysis, information on the choice of priors and Markov chain Monte Carlo settings
☐	☒	For hierarchical and complex designs, identification of the appropriate level for tests and full reporting of outcomes
☐	☒	Estimates of effect sizes (e.g. Cohen's *d*, Pearson's *r*), indicating how they were calculated

Our web collection on statistics for biologists contains articles on many of the points above.

Software and code

Policy information about availability of computer code

Data collection	Living Image IVIS 4.3 software, Seahorse Wave Desktop 2.6 software, Zeiss LSM 800 microscope ZEN 2.6 software, Olympus Cellsens software 2.3, FACSDiva 8.0.2.
Data analysis	Graph Pad 7.0, ELDA (Extreme Limiting Dilution Analysis), ImageJ 1.52p, Seahorse Wave Desktop 2.6 software, Living Image IVIS 4.3 software, FlowJo V10.

For manuscripts utilizing custom algorithms or software that are central to the research but not yet described in published literature, software must be made available to editors and reviewers. We strongly encourage code deposition in a community repository (e.g. GitHub). See the Nature Research guidelines for submitting code & software for further information.

Data

Policy information about availability of data

All manuscripts must include a data availability statement. This statement should provide the following information, where applicable:

- Accession codes, unique identifiers, or web links for publicly available datasets
- A list of figures that have associated raw data
- A description of any restrictions on data availability

All data generated or analyzed during this study are included in the main and supplementary figures. Complete raw data are available from the corresponding author upon request.

Field-specific reporting

Please select the one below that is the best fit for your research. If you are not sure, read the appropriate sections before making your selection.

- [x] Life sciences
- [ ] Behavioural & social sciences
- [ ] Ecological, evolutionary & environmental sciences

For a reference copy of the document with all sections, see nature.com/documents/nr-reporting-summary-flat.pdf

Life sciences study design

All studies must disclose on these points even when the disclosure is negative.

Sample size	Sample size is based on previous publications, which is the most optimal to generate statistically significant results. All experiments were carried out at least three times. For each experiment, n=3 biologically independent samples unless otherwise stated.
Data exclusions	No data exclusions.
Replication	Experiments were performed at least in 3 independent biological replicates and data were reproducible.
Randomization	The samples/cells as well as the mice used in each experiments were randomized.
Blinding	Blinding was applied on all counts and quantifications.

Reporting for specific materials, systems and methods

We require information from authors about some types of materials, experimental systems and methods used in many studies. Here, indicate whether each material, system or method listed is relevant to your study. If you are not sure if a list item applies to your research, read the appropriate section before selecting a response.

Materials & experimental systems

n/a	Involved in the study	
[ ]	[x]	Antibodies
[ ]	[x]	Eukaryotic cell lines
[x]	[ ]	Palaeontology and archaeology
[ ]	[x]	Animals and other organisms
[x]	[ ]	Human research participants
[x]	[ ]	Clinical data
[x]	[ ]	Dual use research of concern

Methods

n/a	Involved in the study	
[x]	[ ]	ChIP-seq
[ ]	[x]	Flow cytometry
[x]	[ ]	MRI-based neuroimaging

Antibodies

Antibodies used

Immunoblotting antibodies: OSMR (1:100, Santa Cruz, #8494), mtHSP70 (1:1000, Invitrogen, #MA3-028), TIM44 (1:500, Abcam, #244466), TOM20 (1:1000, Cell Signaling, #42406), H3K4me3 (1:1000, Abcam, #8580), BCL2 (1:1000, Cell Signaling, #15071), prohibitin (1:1000, Cell Signaling, #2426), NDUFS1 (1:3000, Abcam, #169540), NDUFS2 (1:4000, Abcam, #110249), α-tubulin (1:5000, Abcam, #4074), Na+/K+ ATPase (1:1000, Abcam, #58475), calnexin (1:1000, Abcam, #22595), phospho-Akt (Ser473) (1:1000, Cell Signaling, #4060), phospho-p44/42 MAPK (Thr202/Tyr204) (1:1000, Cell Signaling, #9101) and phospho-STAT3 (Tyr705) (1:1000, Cell Signaling, #9138).
Immunoprecipitation antibodies: (Abnova, #H00009180-B01P), mouse IgG (Millipore, #12-371).
Immunofluorescence antibodies: ATPIF1 (1:100, Invitrogen, #A-21355), OSMR (1:50, Abnova, #H00009180-D01P), secondary Alexa fluor 488 goat anti-rabbit (1:500, Cell Signaling, #4412s) and secondary 594 goat anti-mouse (1:500, Cell Signaling, #8890).

Validation

All the used antibodies are validated by the manufacturers/previous research teams as well as our team.
Immunoblotting antibodies:
-OSMR (1:100, Santa Cruz, #271695, clone D-10): validated for detecting murine and human endogenous protein by WB. Referenced for WB in 2 publications.
Additional validation was performed by ourselves using OSMR CRISPR and control samples.
-mtHSP70 (1:1000, Invitrogen, #MA3-028): detects mtHSP70 from human and mouse tissues. By Western blot, this antibody detects a single ~75 kDa band representing mtHSP70 from U2OS cell homogenate. Reference for WB in 77 publications.
Immunocytochemical staining of mtHSP70 in DAP.3 cells with MA3-028 results in a worm-like staining pattern, consistent with mitochondrial localization. Referenced for immunofluorescence in 8 publications.
Additional validation was performed by ourselves using simtHSP70-treated samples.
-TIM44 (1:500, Abcam, #244466): validated for detecting murine and human endogenous protein by WB.
Additional validation was performed by ourselves using siTIM44-treated samples.
-TOM20 (1:1000, Cell Signaling, #42406): validated for detecting human endogenous protein by WB and recommended for detection

of TOM20 of mouse origin by WB.
Referenced in 21 publications.
-H3K4me3 (1:1000, Abcam, #8580): recommended for detection of H3K4me3 of mouse and human origin by WB.
Referenced in 1455 publications.
-BCL2 (1:1000, Cell Signaling, #15071): validated by WB in Bcl-2 knockout human cells (HeLa).
Referenced in 146 publications.
-prohibitin (1:1000, Cell Signaling, #2426): validated for detecting murine and human endogenous protein by WB.
Referenced in 26 publications.
-NDUFS1 (1:3000, Abcam, #169540): validated for detecting human endogenous protein by WB and immunofluorescence.
Recommended for detection of NDUFS1 of mouse origin by WB.
Referenced in 10 publications.
-NDUFS2 (1:4000, Abcam, #110249): validated by WB in NDUFS2 knockout human cells (HAP1). Validated for detecting murine endogenous protein by WB. Validated for detecting human endogenous protein by immunofluorescence.
Referenced in 6 publications.
-α-tubulin (1:5000, Abcam, #4074): validated for detecting murine and human endogenous protein by WB.
Referenced in 248 publications.
-Na+/K+ ATPase (1:1000, Abcam, #58475): validated for detecting human endogenous protein by WB. Recommended for detection of Na+/K+ ATPase of mouse origin by WB.
Referenced in 15 publications.
-calnexin (1:1000, Abcam, #22595): validated by WB in calnexin knockout human cells (HAP1). validated for detecting murine endogenous protein by WB.
Referenced in 185 publications.
-phospho-Akt (Ser473) (1:1000, Cell Signaling, #4060): validated for detecting human endogenous protein by WB.
Referenced in 3527 publications.
-phospho-p44/42 MAPK (Thr202/Tyr204) (1:1000, Cell Signaling, #9101): recommended for detection of phospho-p44/42 MAPK (Thr202/Tyr204) of mouse human origin by WB.
Referenced in 4089 publications.
-phospho-STAT3 (Tyr705) (1:1000, Cell Signaling, #9138): validated for detecting human endogenous protein by WB.
Referenced in 113 publications.

Immunoprecipitation antibodies:
-OSMR (Abnova, #H00009180-B01P): validated for detecting human endogenous protein by WB. Used for immunoprecipitation in Jahani-Asl et al., Nature Neurosciences, 2016.
-mouse IgG (Millipore, #12-371): validated for use in immunoprecipitation and WB.

Immunofluorescence antibodies:
-ATPIF1 (1:100, Invitrogen, #A-21355): validated for detecting human endogenous protein by immunofluorescence.
Referenced in 2 publications for immunofluorescence.
-OSMR (1:100, Abnova,#H00009180-D01P): validated for detecting human endogenous protein by immunofluorescence.
-secondary Alexa fluor 488 goat anti-rabbit (1:500, Cell Signaling, #4412s): validated for use in immunofluorescence.
Referenced in 212 publications.
-secondary 594 goat anti-mouse (1:500, Cell Signaling, #8890):): validated for use in immunofluorescence.
Referenced in 30 publications.

Eukaryotic cell lines

Policy information about cell lines

Cell line source(s)	The human BTSC line 112, 145, and 172 were generously provided by Dr. Keith Ligon at Harvard Medical School. BTSC12, 73, and 147 were provided by Dr. Samuel Weiss at the University of Calgary.
Authentication	All lines used in this study are characterized in Jahani-Asl et al 2016, Nature Neuroscience.
Mycoplasma contamination	All lines were confirmed negative for mycoplasma using a PCR mycoplasma detection kit (ABM, #G238).
Commonly misidentified lines (See ICLAC register)	No misidentified lines used in this study.

Animals and other organisms

Policy information about studies involving animals; ARRIVE guidelines recommended for reporting animal research

Laboratory animals	7-week-old male SCID mice, 6-7 days old males and females CD1 mouse pups, 2-, 6- and 9-month-old male C57BL/6J mice.
Wild animals	The study did not involve wild animals.
Field-collected samples	The study did not involve samples collected from the field.

Ethics oversight	The use of mice was following ethical regulations and was approved by the McGill University Animal Care Committee (UACC).

Note that full information on the approval of the study protocol must also be provided in the manuscript.

Flow Cytometry

Plots

Confirm that:

- [x] The axis labels state the marker and fluorochrome used (e.g. CD4-FITC).
- [x] The axis scales are clearly visible. Include numbers along axes only for bottom left plot of group (a 'group' is an analysis of identical markers).
- [x] All plots are contour plots with outliers or pseudocolor plots.
- [x] A numerical value for number of cells or percentage (with statistics) is provided.

Methodology

Sample preparation	BTSC were dissociated into single cells using Accumax and stained as indicated in the Methods.
Instrument	BD FACSAria Fusion Cell Sorter, BD FACS CantoII.
Software	BD FACSDiva™ software and data were analyzed using the FlowJo software.
Cell population abundance	10.9% and 16.5%.
Gating strategy	Cell debris were excluded using the FSC-A and SSC-A parameters. Doublets were exluded using the SSC-W and SSC-H parameters. For cell sorting, live cells were DAPi-negative.

- [x] Tick this box to confirm that a figure exemplifying the gating strategy is provided in the Supplementary Information.
